# Can periodontal disease affect conception? A literature review

**DOI:** 10.1530/RAF-20-0043

**Published:** 2021-02-05

**Authors:** Francesco Saverio Ludovichetti, Anna Giulia Signoriello, Edoardo Alvise Gobbato, Anna Artuso, Edoardo Stellini, Sergio Mazzoleni

**Affiliations:** 1 Neurosciences Department – Dentistry Section, Università degli Studi di Padova, Padova, Italy

**Keywords:** periodontal disease, infertility, systemic health

## Abstract

Today periodontal health is considered as an integral part of systemic health itself and no longer as a single factor. The literature recognizes that the presence of periodontal disease can represent a risk factor for numerous systemic conditions such as heart disease and diabetes. In recent years, we have witnessed a progressive interest regarding the influence exerted by this condition on reproduction, as well as the possible repercussions on conception possibilities. Upon analyzing a limited number of studies available for the correlation between periodontal disease and female infertility, it could be inferred that this condition can be equated to the presence of a real outbreak of infection and therefore exert its influence, not only through bacterial translocation in the bloodstream, causing the systemic dissemination of pathogens, but also through the production of cytokines and immunoglobulins by inflammatory mediators. This situation limits bacterial growth, but it could cause damage to the fetus, to the reproductive system, and could hinder conception attempts. Although further research is needed to better clarify the mechanism underlying the possible correlation between periodontal disease and female infertility, the present article aims to review all the available literature on this topic.

**Lay summary:**

In recent years, interest regarding the influence of gum disease on conception has increased. Since serious gum disease (periodontitis) can be compared to an outbreak of infection, studies suggest that the bacteria that mediate inflammation do not remain confined only to the gum tissue, but can enter the bloodstream and spread, thus spreading the infection and having a whole-body effect. This situation could cause damage to the developing baby, to the reproductive system and could hinder conception attempts. Constant maintenance of oral health is definitely necessary. It is important for the professionals involved (gynecologists, obstetricians, dentists, etc.) to communicate and collaborate on these issues. Dentists could advise on the correct hygienic maintenance not only to pregnant women, but also to those who are planning a pregnancy in order to avoid the occurrence of unfavorable conditions.

## Introduction

Periodontal disease or periodontitis can be defined as a chronic degenerative disease with a bacterial etiology that leads to a gradual loss of the support structure of the tooth, such as the gengiva, periodontal ligament, cementum and alveolar bone. To date, this disease represents the main cause of the loss of dental elements in the world (Singh *et al*. 2019).

Severe forms of periodontitis affect a limited population in industrialized countries, about 10–15%, but its incidence increases with age. The peak is around 50–60 years of age (Papapanout & Lindhe 2016).

In recent years, it has been assessed that periodontitis derives from the complex interactions between the host defense mechanisms and the bacterial compounds contained in the biofilm.

The bacterial plaque acts damagingly on two fronts: on one hand, there are damages caused by the direct action of the bacteria and their leukotoxins and enzymes which destroy the defense cells and the connective tissue. On the other hand, there is the inflammatory response triggered by the host and caused by the persistence of the plaque, which leads to the gradual destruction of involved tissues (Cortesi 2017).

However, its progression and severity can also be influenced by other factors: genetic predisposition, lifestyles, general health status (uncompensated diabetes), obesity, malnutrition, osteoporosis and osteopenia, psychosocial stress, hormonal changes, drug assumption, hematological or genetic disorders and other syndromes associated with the category ‘periodontitis as a manifestation of systemic diseases’ (Lalla & Papapanou 2017). The International Committee for Monitoring Assisted Reproductive Technology and the World Health Organization (WHO) define infertility as a disorder of the reproductive system characterized by the inability to establish a natural pregnancy following regular unprotected or targeted relationships after a period of 12 months or more. This condition affects 10–15% of women while in the remaining 80–85%, natural conception occurs within the year. It is strongly influenced by age and lifestyle and it is most frequently found around 30–40 years of age and in particular in women. Moreover, Infertility is often associated with dysfunctions of the ovulatory process, disorders of cervical mucus production, endometriosis, adhesions present at the level of the endometrium, and the presence of particular anatomical conditions affecting the fallopian tubes or the uterus itself (Khanna *et al*. 2017). This disorder is also frequently related to a high risk of adverse pregnancy outcomes, for example premature birth, low birth weight of the newborn and episodes of pre-eclampsia (Martelli *et al*. 2017). Oral biofilm may be responsible for a ‘systemic impairment’ of an individual's health, due to the high inflammatory markers found in patients affected by periodontal disease. This correlation is given by the fact that the bacteria that underlie periodontitis and the mediators of inflammation do not remain confined only to the periodontal tissue, but can enter the bloodstream and spread, thus disseminating the infection and having a systemic effect (Miragliotta & Mosca 2017).

Supporting this hypothesis, the placenta and amniotic fluid may represent a real target for all those bacterial species and for their toxins which, in case of untreated periodontitis, could spread through the bloodstream and therefore become a cause of poly-abortion, preterm births, low birth weight, even going so far as to invalidate attempts at assisted reproduction with a consequent significant economic and biological cost.

There are two mechanisms that would seem to be involved in this scenario: the first one is the dissemination of bacteria from the oral cavity through the bloodstream (Khanna *et al*. 2017, Martelli *et al*. 2017); to support this hypothesis, specimens of *Porphyromonas gingivalis* and *Fusobacterium nucleatum* found in some samples of amniotic fluid or placenta taken from mothers who had a premature birth and who were affected at the same time by periodontitis, and specimens of *P. gingivalis* and *Aggregatibacter actinomycetemcomitans* detected into the liquid amniotic of pregnant women affected by periodontal disease (Martelli *et al*. 2017). The second one is the production of cytokines and immunoglobulins which, despite limiting the growth of pathogenic microorganisms, could however cause harm to the fetus in case of already established pregnancy (Khanna *et al*. 2017, Martelli *et al*. 2017).

Based on these considerations and on the high number of failures that occur during infertility treatment, the scientific community has started to consider other health parameters such as maternal periodontal health from the moment of conception. This is partly due to the high percentage of infertility treatments that still fail today, despite the continuous improvements of the procedures (Khanna *et al*. 2017).

The purpose of the present review is to analyze the available literature in order to investigate whether the presence of periodontal disease may affect female conception.

## Materials and methods

### Types of studies

After a careful selection, we considered 19 articles. Among them, 7 were observational studies, 4 literature reviews, 3 surveys, 2 case–control study, 1 letter to the editor, 1 pilot study and 1 case report. Table 1 summarizes the main characteristics of the included studies.

**Table 1:** Literature articles included in the present review, with their main features and purposes, ordered by date.

Reference	Country	Aims/purpose	Methodology	Key findings and results
Shahryar *et al*. (2009)	USA	To verify the existence of a correlation or reciprocal influence between endometriosis and periodontitis	Survey	The study concludes that a correlation between these two conditions may exist due to the lack of immune regulation (e.g. caused by infectious agent)
Klinger *et al*. (2011)	Israel	To examine the association between fertility parameters and the periodontal status of men attending fertility and* in vitro* fertilization clinic	Observational study	Possible association between periodontal disease, low semen quality and male infertility
Hart *et al*. (2012)	Australia	To verify the influence of periodontitis on the time of female conception	Observational study	Periodontal disease may represent a risk factor for the increase in conception time, especially in non-caucasian women (+2 months)Need for further investigations
McKinnon *et al*. (2013)	Canada	Presentation of a case report about pelvic inflammation due to *Fusobacterium nucleatum*	Case report	Translocation and diffusion of *Fusobacterium n* *ucleatum* through the blood route could lead to the establishment of reproductive health problems in the long term
Nwhator *et al*. (2013)	Nigeria	To verify the level of knowledge about the correlation between periodontal and reproductive health among Nigerian doctors	Survey	Lack of knowledge about the correlation between oral and reproductive health
Nwhator *et al*. (2014)	Nigeria	To investigate the relationship between periodontitis and male infertility	Observational study	A link between poor oral hygiene and low sperm count have been found
Akcali *et al*. (2014)	Turkey	To investigate the level of periodontal pathogens in saliva and of antibody response in serum in subjects with polycystic ovary syndrome	Observational study	Polycystic ovary syndrome may affect the oral microbiota composition and the antibody response to certain members of the microbial community
Umeizudike *et al.* (2016)	Nigeria	To assess Nigerian doctors knowledge regarding the possible correlation between periodontal disease and systemic health	Survey	Lack of knowledge about the theme in study and need for post-diploma training courses
Paztor *et al*. (2016)	Hungary	To evaluate the correlation between poor sperm analysis results of male with idiopathic infertility and periodontitis	Observational study	Pathospermia and poor semen quality seem to be not associated with periodontal infection in men with idiopathic infertility
Prager *et al*. (2017)	Hungary	To study the association between sperm pathology in idiopathic male infertility and periodontal/ dental status	Observational study	Poor periodontal status was associated with oligo and asthenozoospermia
Khalife *et al*. (2019)	Lebanon	To study the correlation between periodontal disease and poor* in vitro* fertilization treatment results	Pilot study	No correlation was found between poor oral hygiene and outcomes of fertilization tratmentsNevertheless, periodontal health is an important factor for general health
Chidambar *et al*. (2019)	India	To investigate the relationship between fertility parameters and periodontal status of male patients	Observational study	A relationship between male infertility, decreased semen quality and periodontal disease seems to have been found
Tao *et al*. (2020)	China	To compare the prevalence of periodontal disease between subjects with semen abnormalities and subjects with normozoospermia	Case–control study	Periodontal disease in correlated with semen abnormalities and sperm motility
Machado *et al*. (2020*a*)	Portugal	To compare the periodontal status among females referred to a fertility clinic with the one of a regional epidemiological sample	Case–control study	Females with searching for fertility treatments presented worse periodontal conditions

### Types of participants

Female and male with periodontitis and infertility.

### Types of interventions

Literature was analyzed searching for a possible connection between infertility and the presence of periodontits.

### Search methods for identification of studies

This literature review was performed formulating the following research question: What literature exists to explain a possible connection between periodontitis and infertility? A comprehensive literature search was completed for the keywords periodontal, disease, infertility, periodontitis, female, male using the Boolean operators ‘and’ and ‘or’ to identify combinations of these words, and limiting the search to the English language and to articles published in the last 10 years with at least two authors. Searches were completed in Medline and Scopus databases.

The authors performed independent title and abstract review to include or eliminate potentially eligible articles. Then, independent full article reviews were performed on the articles included from the initial search to evaluate their relevance to the research question.

### Data collection

After exclusion for duplicates and title, the abstract review allowed considering 23 items, and the full-text reading reduced the number to 19 articles.

## The mechanism of action of periodontitis and its effect on systemic health

Periodontal disease can be considered as a chronic inflammatory condition that includes gingivitis, a mild and reversible form that affects the soft tissues around the dental elements, and periodontitis, a severe form that culminates in the destruction of soft tissues, alveolar bone and other dental support structures (Kavoussi *et al*. 2009).

Specific anaerobic bacteria present inside the periodontal pockets are believed to be the main cause for the development of the more severe form of periodontal disease (Hart 2012, Hart *et al.* 2012, Akcali *et al*. 2014, Khanna *et al*. 2017, Martelli *et al*. 2017, Khalife *et al*. 2019). The activation of the host response, triggered by these microorganisms and their metabolites, stimulates the synthesis and release of cytokines, mediators of inflammation and metalloproteinases which then contribute to tissue destruction. The progression and severity of the disease will depend on the balance between the aggressiveness of subgingival biofilm and the individual immune response, with possible influences from the genetic and epigenetic context, and from some environmental factors such as sex, age, smoking habit and the degree of oral hygiene (Martelli *et al*. 2017). Once the infection is established, a specific cascade of destructive tissue mechanisms is activated and triggered by the inflammation mediators. It is also possible that these molecules, as well as the pathogens involved in the development of the disease, do not remain confined to the periodontal tissue but reach the bloodstream and disseminate the infection (Hart *et al.* 2012, Martelli *et al*. 2017).

During the 19th century and the beginning of the 20th century, there was the spread of the ‘focal theory’, which states that the presence of outbreaks of infections could influence the onset or progression of a wide variety of inflammatory disorders such as arthritis or heart problems. In this regard, more than 200 microbial species have been identified in apical periodontitis and more than 500 in its marginal variant. Since bacteremia in humans can reach very high levels (more than 1011 microorganisms per mg of dental plaque) and considering that these pathogens are close to the circulatory system, it is easy to understand how the systemic spread of bacterial products, as well as derivatives of the inflammatory response, is facilitated by these conditions (Khanna *et al*. 2017). In support of these considerations, some studies have also suggested an alteration of inflammatory markers after a successful periodontal treatment, with a reduction in the levels of c-reactive protein and interleukins, and with an improvement in vascular and endothelial functions (Hart *et al.* 2012). Furthermore, other studies have found high levels of IgG antibacterial serum antibodies in patients who have a chronic or aggressive form of periodontitis, compared to those present in individuals with good oral health and without early signs of periodontal disease. However, the presence of serum antibodies against periodontal pathogens does not confer immunity toward them and does not prevent the development of the disease, but is considered an auxiliary practice for its diagnosis (Akcali *et al*. 2014). Due to the possibility of systemic dissemination of periodontal infection through the bloodstream, clear evidence indicates the presence of a correlation between oral and systemic health. Much of the literature recognizes that periodontal disease may represent a risk factor for numerous systemic conditions (including female infertility). In the last 20 years, there has been a constant development of the so-called ‘periodontal medicine’, which today is considered a very important branch of periodontal clinical research. For this reason, the maintenance of a good level of oral health is always encouraged in order to prevent or mitigate the course of a variety of conditions (Martelli *et al*. 2017).

## Periodontitis and female infertility

The presence of a correlation between periodontits and female infertility is not yet well defined. Many attempts have been made by the scientific community to understand if an association between these two conditions exists.

In 2012, Hart *et al*., after analyzing the clinical parameters of 3416 women, postulated that, the presence of periodontitis could represent a risk factor for female conception (especially in non-Caucasian women considering the high frequency of periodontitis, found in 41.4% of the sample). In a randomized controlled multicenter study (Smile Study) conducted in Australia, an average conception time of 7.1 months in women presenting oral inflammation (mostly non-Caucasian) against the value of 5 months was found in women having a good oral health. Moreover, they detected an increase of over 2 months on the average female conception time in non-Caucasian women compared to the others (Hart *et al*. 2012).

A study conducted in Nigeria by Umeizudike *et al*. in 2016, underlined considerable gaps on this issue, thus highlighting the essential need of supporting the attendance of postgraduate courses by health professionals, with a view to continuous training (Umeizudike *et al*. 2016). The present work confirmed the results previously obtained (2013) by Nwhator *et al*. for which only 17.6% of the participants (119 professionals among doctors, specialists and doctors in specialized training) recognized periodontitis a risk factor for fertility, however, the low number of subjects involved in the work should be considered to interpret these results (Nwhator *et al*. 2013).

In the last period, it was possible to record an increase in interest about the link between periodontal disease and female infertility, although further investigation is needed.Martelli *et al*. (2017). In support of this consideration, the article published by McKinnon *et al*. in 2013, where was reported a pelvic inflammation caused by *F. nucleatum*, an oral pathogen, the case was called atypical because the young protagonist had never had full sexual relations (McKinnon *et al*. 2013).

As reported by Martelli *et al*. (2017), there are two possible mechanisms underlying this phenomenon. The first one is the achievement of the circulatory system by the pathogens and their consequent diffusion through it (Khanna *et al*. 2017, Martelli *et al*. 2017). In support of this, samples of *P. gingivalis* and *F. nucleatum* were identified in the amniotic fluid or in the placenta of mothers having had a premature birth and simultaneously presenting periodontitis; or samples of *P. gingivalis* and *A. actinomycetemcomitans* were identified in the amniotic fluid of future mothers with periodontitis. The bacteremia created inside the uterus, as a consequence of endotoxins and bacterial products, could cause alterations in fetus development and in conception (Khanna *et al*. 2017). The second one is the activation of the immune response by the host with the production of cytokines and immunoglobulins, which, if on one hand counteracts the growth of pathogens, and on the other hand, could also cause damage to the fetus or to the reproductive system itself (Khanna *et al*. 2017, Martelli *et al*. 2017).

Despite this, the review published by Machado *et al*. in 2020 would seem to highlight no connection between the presence of periodontitis and the presence of infertility-related conditions (Machado *et al*. 2020*b*) contrasting with the results of another study, at the end of which it was not entirely excluded that the presence of periodontitis may be a risk factor for reproductive health (Ludovichetti *et al*. 2020).

The results of the following review also showed that recently, there also been an attempt to identify the presence of a link between periodontal disease and polycystic ovary syndrome or endometriosis, two conditions often associated with infertility (Kavoussi *et al*. 2009, Akcali *et al*. 2014).

Polycystic ovary syndrome is a condition considered as one of the main causes of female infertility. It is a hormonal disorder affecting women of reproductive age with a range from 6.5 to 8% and it is characterized by the presence of low-grade chronic inflammation (Akcali *et al*. 2014). As mentioned earlier, in the study published in 2006, Hart *et al*. suggested how this condition could be improved not only through the improvement of lifestyle and diet, but also through the administration of metformin, the long-term effects of which still remain to be clarified (adverse cardiovascular outcomes, miscarriage, gestational diabetes, pre-eclampsia and fetal macrosomy) (Hart *et al*. 2007).

In a study published in 2014, Akcali *et al*. hypothesized that the presence of this condition may influence the composition of the oral microbiota, raise the immune response against some bacteria present in this microbial community and then determine repercussions on periodontal health. The work aimed to detect seven periodontal pathogens in saliva and the presence of the respective serum antibodies. In this case, it was also hypothesized that this correlation could also be enhanced by a disorder in the systemic inflammatory response (Akcali *et al*. 2014). In support of this hypothesis, a study published by Weiss *et al*., would also report as the presence of inflammation, although on the one hand necessary for the presence of optimal fertility and good tissue remodeling, on the other hand, it could represent an obstacle to endometrial receptivity and trophoblast-endometrium interaction (Weiss *et al*. 2009).

The dual influence that the two conditions could have, one against the other (PCOS and periodontal disease) is also assumed by Işık *et al*. at the end of whose study, targeted for detection of periodontal status in women with different PCOS phenotypes, high pocket depth values were detected in all different types of polycystic ovary (Işık *et al.* 2020)

Endometriosis is a condition that affects about 6–10% of women of reproductive age and is characterized by the presence of glands and endometrial stroma outside the uterine cavity. It is considered as one of the potential causes of pelvic pain and infertility. Regarding periodontal disease, endometriosis is thought to be a very common chronic inflammatory process, especially in people affected by an autoimmune disorder. Kavoussi *et al*. concluded that women with endometriosis presented an enhanced probability of developing periodontitis with respect to healthy women and assumed the existence of a mutual influence between the two conditions. In fact, according to the authors, this connection could derive from a lack of global regulation of the immune response, particularly because of the presence of an inflammatory process in both conditions. However, it is necessary to clarify that the tool used in the present study (National Health and Nutrition Examination Survey) was not designed specifically to meet the purpose of the present work (Kavoussi *et al*. 2009). Recently, as mentioned earlier, the scientific community has begun to consider other health parameters such as maternal periodontal health from the moment of conception. This is partly due to the high percentage of infertility treatments that still fail today, despite the continuous improvements of the procedures. Even if the data with which we must confront are not very numerous and they are often conflicting with each other, Khanna *et al*. in 2017 described a study conducted in 2013 by Pavlatou *et al*. where a correlation was reported between the number of oocytes obtained following ovarian stimulation and the mother’s periodontal health before a treatment. On that occasion, it was also highlighted that maternal periodontium could be affected by hormonal therapies administered before treatments and this could have an impact on the successful outcome of the therapy (Khanna *et al*. 2017). In 2020, Machado *et al*. evaluated the periodontal status between females referenced for fertility treatment emphasizing that females referenced for fertility treatment presented worse periodontal measures than females from a representative control sample (Machado *et al*. 2020*b*) These results were in contrast to the study of Khalife *et al*. (2019) where a correlation was not found between maternal periodontal health before treatments and IVF’s outcomes. In the present study, 28 women and their periodontal indexes were examined before and after treatment. None were found to be affected by periodontitis; 17 women (60.7%) had a positive pregnancy test result with a final total of 13 births (40.6%) and 4 unsuccessful pregnancies (14.3%). The results of the study showed no significant differences between the average values of the two groups, with a positive or an unsuccessful outcome of the *in vitro* fertilization. Nevertheless, the low number of women involved in the study could represent a weak point (Khalife *et al*. 2019).

Based on the earlier studies, it is important to underline how, even if the majority of gynecologists agree that an oral screening can actually be included in the prenatal care program, most of them do not examine the oral cavity of their patients or do not refer them to a specialist dental examination. For this reason, it would be important that an oral health examination becomes an integral part of the prenatal care program, as well as in the case of infertility treatments in order to ‘educate’ couples on the importance of maintaining good oral health in order to remove any probable outbreak of infection with a view to preventing the occurrence of unpleasant conditions (Khanna *et al*. 2017).

## Periodontitis and male infertility

It has been estimated that more than 48.5 million couples suffer from infertility and about 40% of these could be attributed to male infertility (Khanna *et al*. 2017). In recent years, various authors have tried to correlate the presence of poor oral hygiene with the presence of male infertility. It has been established that >30% of male infertility cases are of idiopathic etiology (Chidambar *et al*. 2019). In a study published in 2014 by Nwhator *et al*., a significant correlation was highlighted between the presence of periodontitis and sperm count. Out of 76 subjects analyzed, 55 showed an abnormal sperm count (Nwhator *et al*. 2014). The results of the present study proved to be in agreement with those of another work published by Klinger *et al*. in 2011 that evaluated the fertility parameters of male subjects attending *in vitro* fertilization with the purpose of examining their periodontal health. At the end of that work, the presence of a correlation between periodontitis, low sperm quality and periodontal situation in reference to these subjects in expectation to undergo *in vitro* fertilization was not excluded. On that occasion, 88% of the participants were found to be suffering from gingivitis or periodontitis and 63% of the total turned out to have an abnormal sperm count (Klinger *et al*. 2011, Khanna *et al*. 2017). Furthermore, in accordance with these considerations, other works (Prager *et al*. 2017, Kellesarian *et al*. 2018, Chidambar *et al*. 2019, Tao *et al*. 2020) also highlighted a possible link between poor oral hygiene and sperm abnormalities. Pràger *et al*. in 2017 showed that the extent of gum bleeding and the presence of BOP or calculus could be associated with oligospermia in subjects with idiopathic male infertility (Prager *et al*. 2017). These results, however, were in contrast to those of a work published in 2016 by Pàsztor *et al*. at the end of which no type of correlation was highlighted between these two conditions (periodontitis and male idiopathic infertility), and poor periodontal status was not related with any sperm pathology parameters. (Pasztor *et al*. 2016).

In 2018, Kellesarian *et al*. conducted a literature review, at the end of which the presence of a link between periodontal disease and the presence of male infertility emerged as an eventuality feared by numerous works highlighting the need for further studies and insights (Kellesarian *et al*. 2018) The results of the present work were therefore found to be in accordance with Chidambar *et al*. (2019) at the end of which a correlation was found between the number of sites with high loss of clinical attachment and mobility and sperm counts (Chidambar *et al*. 2019).

In 2020, Tao *et al*. found that male subjects with periodontal disease were more likely to experience abnormalities at the sperm level. The case group, whose participants had some semen abnormality, had a higher incidence of moderate or severe periodontitis compared to the control group (Tao *et al*. 2020).

## Conclusions

In conclusion, the analysis of the cited articles shows that the information obtained is considerable, but not yet sufficient to establish with certainty the presence of a link between periodontitis and female/male infertility.

Therefore, what emerges from the following review is the need for many other studies in order to obtain more data, even if there is no doubt that oral health does influence the quality of life of an individual, and that it is no longer considered separately, but an integral part of it. A constant maintenance of a good degree of oral health is definitely necessary.

The updating and the presence of collaboration between the various professionals involved (gynecologists, obstetricians, dentists, etc.) are important. Dentists could advise on the correct hygienic maintenance, both professional and at home, not only to the pregnant women, but also to those who are planning a pregnancy in order to avoid the occurrence of unfavorable conditions.

## Declaration of interest

The authors declare that there is no conflict of interest that could be perceived as prejudicing the impartiality of the research reported.

## Author contribution statement

F S L was involved with the conceptualization, design, definition of intellectual content, manuscript review; A G S was involved in manuscript preparation, manuscript editing; E A G was involeved in manuscript review; A A was invloved in manuscript preparation, E S and SM were involved inmanuscript review.

## References

[bib1] Akcalı A Bostanci N Özçaka Ö Öztürk-Ceyhan B Gümüş P Buduneli N Belibasakis GN 2014 Association between polycystic ovary syndrome, oral microbiota and systemic antibody responses. PLoS ONE 9 e108074. (10.1371/journal.pone.0108074) PMC4169459 25232962

[bib2] Chidambar CK Shankar SM Agarwal RK Bhushan KS Gururaj SB 2019 Evaluation of periodontal status among men undergoing infertility treatment. Journal of Human Reproductive Sciences 12 130–135. (10.4103/jhrs.JHRS_168_18) 31293327 PMC6594118

[bib3] Cortesi AV 2017 Malattie del parodonto: gengivite e parodontite in. In Igienista orale teoria e pratica professionale. Eds Cortesi AV Abbinante. AA cura di: Milano: Edra S.p.A., pp. 159–181.

[bib4] Hart R 2007 Polycystic ovarian syndrome--prognosis and treatment outcomes. Current Opinion in Obstetrics and Gynecology 19 529–535. (10.1097/GCO.0b013e3282f10e22) 18007129

[bib5] Hart R 2012 Periodontal disease: could this be a further factor leading to subfertility and is there a case for a prepregnancy dental check-up? Women’s Health 8 229–230. (10.2217/whe.12.15) 22554169

[bib6] Hart R Doherty DA Pennell CE Newnham IA Newnham JP 2012 Periodontal disease: a potential modifiable risk factor limiting conception. Human Reproduction 27 1332–1342. (10.1093/humrep/des034) 22362927

[bib7] Işık Y Telatar GY Neşelioğlu S Biçer C Gürlek B 2020 Evaluation of periodontal status in different phenotypes of polycystic ovary syndrome in untreated patients of early reproductive age: a case-control study. Journal of Obstetrics and Gynaecology Research 46 459–465. (10.1111/jog.14179) 31922343

[bib8] Kavoussi SK West BT Taylor GW Lebovic DI 2009 Periodontal disease and endometriosis: analysis of the National Health and Nutrition Examination Survey. Fertility and Sterility 91 335–342. (10.1016/j.fertnstert.2007.12.075) 18394619 PMC2674278

[bib9] Kellesarian SV Yunker M Malmstrom H Almas K Romanos GE Javed F 2018 Male infertility and dental health status: a systematic review. American Journal of Men’s Health 12 1976–1984. (10.1177/1557988316655529) PMC6199424 27339766

[bib10] Khalife D Khalil A Itani MN Khalifeh F Faour S Salame A Ghazeeri G 2019 No association between the presence of periodontal disease and poor IVF outcomes: a pilot study. International Journal of Women’s Health 11 363–370. (10.2147/IJWH.S202135) PMC6572705 31354363

[bib11] Khanna SS Dhaimade PA Malhotra S 2017 Oral Health status and fertility treatment including IVF. Journal of Obstetrics and Gynaecology of India 67 400–404. (10.1007/s13224-017-1025-0) 29162952 PMC5676574

[bib12] Klinger A Hain B Yaffe H Schonberger O 2011 Periodontal status of males attending an in vitro fertilization clinic. Journal of Clinical Periodontology 38 542–546. (10.1111/j.1600-051X.2011.01720.x) 21443558

[bib13] Lalla E Papapanou PN 2016 Fattori di modificazione. In Parodontologia clinica e implantologia orale, sesta edizione. Eds Lindhe J Lang. Na cura di. Milano: Edi Ermes, pp. 274–294.

[bib14] Ludovichetti FS Signoriello AG Artuso A Zuccon A Stellini E Mazzoleni S 2020 Periodontitis and female infertility: is there a connection? Oral Diseases. (10.1111/odi.13636). 32890435

[bib15] Machado V Botelho J Proença L Mendes JJ 2020a Comparisons of periodontal status between females referenced for fertility treatment and fertile counterparts: a pilot case-control study. International Journal of Environmental Research and Public Health 17 5281. (10.3390/ijerph17155281) PMC7432159 32707937

[bib16] Machado V Lopes J Patrão M Botelho J Proença L Mendes JJ 2020b Validity of the association between periodontitis and female infertility conditions: a concise review. Reproduction 160 R41–R54. (10.1530/REP-20-0176) 32716008

[bib17] Martelli ML Brandi ML Martelli M Nobili P Medico E Martelli F 2017 Periodontal disease and women’s health. Current Medical Research and Opinion 33 1005–1015. (10.1080/03007995.2017.1297928). 28277873

[bib18] McKinnon A Black AY Lortie K Fleming NA 2013 A case of adolescent pelvic inflammatory disease caused by a rare bacterium: Fusobacterium nucleatum. Journal of Pediatric and Adolescent Gynecology 26 e113–e115. (10.1016/j.jpag.2013.02.008) 23619431

[bib19] Miragliotta G Mosca A 2017 Il biofilm batterico nel cavo orale in Cortesi AV, Abbinante A, Igienista orale teoria e pratica professionale. Milano: Edra S.p.A., pp. 121–126.

[bib20] Nwhator SO Umeizudike KA Ayanbadejo PO Opeodu OI Olamijulo JA Sorsa T 2014 Another reason for impeccable oral hygiene: oral hygiene-sperm count link. Journal of Contemporary Dental Practice 15 352–358. (10.5005/jp-journals-10024-1542) 25307820

[bib21] Nwhator SO Umeizudike KA Samuel TA Soroye MO Umeizudike TI 2013 Periodontitis and sub-fertility; opinions and practices of Nigerian specialists. West African Journal of Medicine 32 267–271. 24488281

[bib22] Papapanou NP Lindhe J 2016 Epidemiologia delle patologie parodontali. In Parodontologia clinica e implantologia orale, sesta edizione. Eds Lindhe J Lang. NA cura di: Milano: Edi Ermes, pp. 129–170.

[bib23] Pásztor N Kárpáti K Szöllősi J Keresztúri M Kozinszky Z Gorzó I Radnai M 2016 Association between periodontal status and idiopathic male infertility. Journal of Oral Science 58 247–253. (10.2334/josnusd.15-0586) 27349547

[bib24] Práger N Pásztor N Várnagy Á Kozinszky Z Baráth Z Gorzó I Radnai M 2017 Idiopathic male infertility related to periodontal and caries status. Journal of Clinical Periodontology 44 872–880. (10.1111/jcpe.12785) 28746727

[bib25] Singh VP Gan JY Liew WL Kyaw Soe HH Nettem S Nettemu SK 2019 Association between quality of sleep and chronic periodontitis: a case-control study in Malaysian population. Dental Research Journal 16 29–35. (10.4103/1735-3327.249555) 30745916 PMC6340225

[bib26] Tao DY Zhu JL Xie CY Kuang YP Chai WR Lo ECM Ye W Li F Feng XP Lu HX 2020 Relationship between periodontal disease and male infertility: a case-control study. Oral Diseases. (10.1111/odi.13552) 32702140

[bib27] Umeizudike KA Iwuala SO Ozoh OB Ayanbadejo PO Fasanmade OA 2016 Association between periodontal diseases and systemic illnesses: a survey among internal medicine residents in Nigeria. Saudi Dental Journal 28 24–30. (10.1016/j.sdentj.2015.03.005) PMC4688436 26792966

[bib28] Weiss G Goldsmith LT Taylor RN Bellet D Taylor HS 2009 Inflammation in reproductive disorders. Reproductive Sciences 16 216–229. (10.1177/1933719108330087) 19208790 PMC3107847

